# Supplementary material for: Vibrio cholerae O1 experiences mild bottlenecks through the gastrointestinal tract in some but not all cholera patients
Source: Microbiol Spectr. 2024 Jun 25;12(8):e00785-24. doi: 10.1128/spectrum.00785-24 (PMC11302224; doi:10.1128/spectrum.00785-24)
Supplement: Supplemental material — Fig. S1 to S2; Tables S1 to S5. [file spectrum.00785-24-s0001.docx]

**TITLE:** *Vibrio cholerae* O1 experiences mild bottlenecks through the gastrointestinal tract in some but not all cholera patients

**AUTHORS:** Patrick Lypaczewski*^1^, Denise Chac*^2^, Chelsea N. Dunmire^2^, Kristine M. Tandoc^2^, Fahima Chowdhury^3^, Ashraful I. Khan^3^, Taufiq Bhuiyan^3^, Jason B. Harris^4,5,6^ , Regina C. LaRocque^5,7^, Stephen B. Calderwood^5,7^, Edward T. Ryan^5,7,8^, Firdausi Qadri^3^, B. Jesse Shapiro^^1^, Ana A. Weil^^2,9^

**AFFILIATIONS:**

^1^Department of Microbiology and Immunology, McGill University, Montreal, QC, Canada

^2^Department of Medicine, University of Washington, Seattle, WA, USA

^3^Infectious Diseases Division, International Center for Diarrheal Disease Research, Bangladesh, Dhaka, Bangladesh

^4^Department of Pediatrics, Massachusetts General Hospital, Boston, MA, USA

^5^Division of Infectious Diseases, Massachusetts General Hospital, Boston, MA, US

^6^Division of Global Health, Massachusetts General Hospital *for* Children, Boston, MA, USA

^7^Department of Medicine, Harvard Medical School, Boston, MA, USA

^8^Department of Immunology and Infectious Diseases, Harvard School of Public Health, Boston, MA, USA

^9^Department of Global Health, University of Washington, Seattle, WA, USA

**SUPPLEMENTARY METHODS**

# *Sample collection and* Vibrio cholerae *isolation*

# All samples were collected **in Dhaka** at the International Centre for Diarrhoeal Disease Research, Bangladesh (icddr,b) following the informed consent process. A maximum of 50 mL of vomit and stool were collected immediately upon admission concurrent with clinical interventions, including rehydration and administration of antibiotics. Samples were immediately frozen at −80 °C.

Vomit and diarrheal stool from Bangladesh were stored at the University of Washington at −80 °C and inoculated directly into alkaline peptone water and streaked onto thiosulfate-citrate-bile salts-sucrose agar, a medium selective for *V. cholerae,* or Luria-Bertani (**LB**) agar and tryptic soy agar containing 5% sheep’s blood.

### *DNA extraction and sequencing*

Bacterial glycerol stocks were streaked on LB agar and incubated at 30 °C for 24 hours, and a single colony was picked and grown in 4 mL LB broth with agitation at 37 °C for 18 hours. Genomic DNA was extracted from each of the 200 isolates and 10 control colonies using the Qiagen DNeasy Blood and Tissue kit with RNAse treatment according to manufacturer's instructions. DNA was then eluted in molecular grade DNase/RNase-free water. Sequencing libraries were prepared with the Lucigen NxSeq AmpFREE kit, pooled and sequenced at the McGill Genome Centre on one lane of Illumina NovaSeq6000 Sprime v1.5 with paired-end 150 bp reads.

**SUPPLEMENTARY TABLES and FIGURES**

##

## Supplementary Figure S1. Breadth of coverage of the MJ-1236 *V. cholerae* O1 reference genome.

## The 200 genomes are shown in arbitrary order along the x-axis, with the y-axis demonstrating the breadth of coverage of the MJ-126 reference genome after mapping short reads. Isolates BSC08, GSC06, HVC01, IVC01, IVC10, JSC08 and JSC11 were >2 standard deviations below the median (94.96%) and were therefore excluded from SNV analysis.

**Supplementary Figure S2. Intra-sample SNVs based on a colony control genome reference*.***

Intra-sample variation based on SNVs called against the colony control assembled genome across paired vomit and stool *V. cholerae* O1 populations demonstrate a decrease in diversity in 4 patients, an increase in 2 patients and no change in 4 patients.

**Supplementary Table S1: Primers used for PCR**

| **Gene target** | **Forward Primer (5' - 3')** | **Reverse Primer (5' - 3')** | **Expected Amplicon (bp)** | **Reference** |
| --- | --- | --- | --- | --- |
| *tcpA* | AGCCGCCTAGATAGTCTGTG | TCGCCTCCAATAATCCGAC | 1200 | (1) |
| *tcpR* | CATGACTAGCATATGGTTACATG | TCACATTAACCAAAATACGCC | 456 | (2) |

**Supplementary Table S2. Comparison of media types used to isolate *V. cholerae* O1 from vomit and stool samples and effects on intra-sample SNVs.**

To maximize isolate yield, we used several media types to isolate *V. cholerae* from clinical samples. We found no difference in the number of intra-sample SNVs based on difference in media used for isolation (Chi-squared value = 9.52, df = 10, *p* = 0.48). **APW** = Alkaline peptone water; **TCBS** = Thiosulfate–citrate–bile salts–sucrose. **TSA** = Tryptic Soy Agar

| **Media Type** | **# of Stool Isolates** | **# of Vomit Isolates** | **Isolates with 0 SNVs** | **Isolates with 1 SNV** | **Isolates with 2 SNVs** |
| --- | --- | --- | --- | --- | --- |
| APW plated onto LB agar | 13 | 18 | 28 | 3 | 0 |
| APW plated onto TCBS agar | 9 | 28 | 33 | 4 | 0 |
| LB agar | 28 | 16 | 42 | 2 | 0 |
| LB broth plated onto LB agar | 14 | 14 | 24 | 2 | 2 |
| LB broth plated onto TCBS agar | 11 | 1 | 11 | 1 | 0 |
| TSA + 5% Sheep’s blood agar | 25 | 23 | 42 | 5 | 1 |

**Supplementary Table S3. SNVs called based on the MJ-1236 *V. cholerae* O1 genome reference.**

The sample types, genes and the SNV effect are shown. Isolates from patients A, B, and G had no intra-sample SNVs. Gene ID and Product annotations are derived from the MJ-1236 *V. cholerae* O1 reference genome.

| **Patient** | **Sample type** | **Gene ID** | **Product** | **SNV** |
| --- | --- | --- | --- | --- |
| C | Stool | VCD_001105 | IS3 family transposase(pseudo) |  |
| D | Stool | VCD_001393 | glycosyltransferase family 9 protein | Arg169fs |
|  | Stool | VCD_002237 | flagellar hook-basal body complex protein FliE | Ser69Thr |
|  | Vomit | VCD_001374 | GDP-mannose 4,6-dehydratase | Thr127fs |
|  | Vomit | VCD_001392 | glycosyltransferase family 2 protein | Cys213Tyr |
|  | Vomit | VCD_003563 | ribosome biogenesis GTPase Der | Ala146Val |
| E | Vomit | VCD_001366 | acyl-CoA reductase | His664Tyr |
| F | Stool | VCD_002976 | 7-carboxy-7-deazaguanine synthase QueE | Pro217fs |
|  | Stool | VCD_001105 | IS3 family transposase (pseudo) |  |
| H | Stool | VCD_000480 | retention module-containing protein | Gly452Asp |
|  | Vomit | VCD_001352 | mannose-6-phosphate isomerase, class I | Thr74fs |
|  | Vomit | VCD_003198 | ATP-dependent Clp protease ATP-binding subunit ClpA | Ala417fs |
| I | Stool | VCD_000299 | phospho-sugar mutase | Pro461Ser |
| J | Stool | VCD_000300 | amino acid ABC transporter permease | Ser177fs |
|  | Vomit | VCD_001362 | glycosyltransferase family 4 protein | Gly232fs |
|  | Vomit | VCD_001362 | glycosyltransferase family 4 protein | Leu301Phe |
|  | Vomit | VCD_001366 | acyl-CoA reductase | Ser97Tyr |
|  | Vomit | VCD_002072 | riboflavin synthase | Ser200Arg |

**Supplementary Table S4. SNVs based on colony control genome reference.**

The patients, source, genes and the effect of the SNV are listed. Isolates from patients A, B, and G had no intra-sample SNVs. Gene ID are based on Prokka annotations of a colony-control genome. Product annotations are derived from the MJ-1236 *V. cholerae* O1 reference genome. *One SNV found here but not in Table S2 is highlighted.*

| **Patient** | **Source** | **Gene ID** | **Product** | **SNV** |
| --- | --- | --- | --- | --- |
| C | stool |  |  | T>A (pseudo) |
| D | Stool | CC_02_02790_gene | glycosyltransferase family 9 protein | Arg169fs |
|  |  | CC_02_01528_gene | flagellar hook-basal body complex protein FliE | Ser78Thr |
|  | vomit | CC_02_00670_gene | ribosome biogenesis GTPase Der | Ala146Val |
|  |  | CC_02_02771_gene | GDP-mannose 4,6-dehydratase | Thr127fs |
|  |  | CC_02_02789_gene | glycosyltransferase family 2 protein | Cys213Tyr |
| E | vomit | CC_02_02763_gene | acyl-CoA reductase | His664Tyr |
| F | stool | CC_02_00089_gene | 7-carboxy-7-deazaguanine synthase QueE | Pro188fs |
|  |  |  |  | TA>T (pseudo) |
| H | stool | CC_02_01149_gene | retention module-containing protein | Gly452Asp |
|  | vomit | CC_02_00302_gene | ATP-dependent Clp protease ATP-binding subunit ClpA | Ala417fs |
|  |  | CC_02_02749_gene | mannose-6-phosphate isomerase, class I | Thr74fs |
| I | Stool | CC_02_00968_gene | phospho-sugar mutase | Pro461Ser |
|  | vomit | CC_02_03009_gene | BREX-1 system phosphatase PglZ type B | Ala272fs |
| J | stool | CC_02_00969_gene | amino acid ABC transporter permease | Ser177fs |
|  | vomit | CC_02_02758_gene | glycosyltransferase family 4 protein | Gly192fs |
|  |  | CC_02_02758_gene | glycosyltransferase family 4 protein | Leu261Phe |
|  |  | CC_02_02763_gene | acyl-CoA reductase | Ser97Tyr |
|  |  | CC_02_01672_gene | riboflavin synthase | Ser200Arg |

**Supplementary Table S5. Presence of genes in *tcp* operon of genomes resequenced with Oxford Nanopore Technologies.**

Presence of *V. cholerae* O1 *tcp* operon genes in the 8 resequenced isolates as determined by panaroo. Genes present are labeled with ‘1’. An asterisk (*) denotes the presence of a frameshift within the gene or 5’ of the gene but affecting the start site of the gene. Frameshifts were always associated with a homopolymer sequence.

| **Isolate** | **tcpJ** | **tcpF** | **tcpE** | **tcpT** | **tcpS** | **tcpD** | **tcpR** | **tcpC** | **tcpQ** | **tcpB** | **tcpA** | **tcpH** | **tcpP** | **tcpI** |
| --- | --- | --- | --- | --- | --- | --- | --- | --- | --- | --- | --- | --- | --- | --- |
| AVC08 | 1* | 1 | 1* | 1 | 1 | 1 | 1 | 1 | 1 | 1 | 1 | 1 | 1 | 1 |
| DVC12 | 1* | 1 | 1 | 1 | 1 | 1 | 1 | 1 | 1 | 1 | 1 | 1 | 1 | 1* |
| GVC10 | 1* | 1 | 1* | 1 | 1 | 1 | 1* | 1 | 1 | 1 | 1 | 1 | 1 | 1 |
| HSC09 | 1 | 1 | 1 | 1 | 1 | 1 | 1 | 1 | 1 | 1 | 1 | 1 | 1 | 1* |
| HVC01 | 1 | 1 | 1 | 1 | 1 | 1 | 1* | 1 | 1 | 1 | 1 | 1 | 1 | 1 |
| IVC01 | 1* | 1 | 1 | 1 | 1 | 1* | 1 | 1 | 1 | 1 | 1 | 1 | 1 | 1 |
| IVC10 | 1* | 1 | 1* | 1 | 1 | 1 | 1 | 1 | 1 | 1 | 1* | 1 | 1 | 1 |
| JSC08 | 1* | 1 | 1* | 1 | 1 | 1 | 1* | 1 | 1 | 1 | 1 | 1 | 1 | 1 |

**Supplementary References**

1. Boyd EF, Moyer KE, Shi L, Waldor MK. 2000. Infectious CTXPhi and the vibrio pathogenicity island prophage in Vibrio mimicus: evidence for recent horizontal transfer between *V. mimicus* and *V. cholerae.* Infect Immun 68:1507–1513.

2. Mishra A, Srivastava R, Pruzzo C, Srivastava BS. 2003. Mutation in tcpR gene (Vc0832) of *Vibrio cholerae* O1 causes loss of tolerance to high osmolarity and affects colonization and virulence in infant mice. J Med Microbiol 52:933–939.
